# Criminal justice pathways to psychiatric care for psychosis

**DOI:** 10.1192/bjp.bp.114.153882

**Published:** 2015-12

**Authors:** Kamaldeep Bhui, Simone Ullrich, Constantinos Kallis, Jeremy W. Coid

**Affiliations:** **Kamaldeep Bhui**, MD, **Simone Ullrich**, PhD, **Constantinos Kallis**, PhD, **Jeremy W. Coid**, MD, Wolfson Institute of Preventive Medicine, Barts & The London School of Medicine & Dentistry, Queen Mary University of London, London, UK

## Abstract

**Background**

Some patients are at higher risk of contact with criminal justice agencies when experiencing a first episode of psychosis.

**Aims**

To investigate whether violence explains criminal justice pathways (CJPs) for psychosis in general, and ethnic vulnerability to CJPs.

**Method**

Two-year population-based survey of people presenting with a first-episode of psychosis. A total of 481 patients provided information on pathways to psychiatric care. The main outcome was a CJP at first contact compared with other services on the care pathway.

**Results**

CJPs were more common if there was violence at first presentation (odds ratio (OR) = 4.23, 95% CI 2.74–6.54, *P*<0.001), drug use in the previous year (OR = 2.28, 95% CI 1.50–3.48, *P*<0.001) and for high psychopathy scores (OR = 2.54, 95% CI 1.43–4.53, *P* = 0.002). Compared with White British, CJPs were more common among Black Caribbean (OR = 2.97, 95% CI 1.54–5.72, *P*<0.001) and Black African patients (OR = 1.95, 95% CI 1.02–3.72, *P* = 0.01). Violence mediated 30.2% of the association for Black Caribbeans, but was not a mediator for Black African patients. These findings were sustained after adjustment for age, marital status, gender and employment.

**Conclusions**

CJPs were more common in violent presentations, for greater psychopathy levels and drug use. Violence presentations did not fully explain ethnic vulnerability to CJPs.

The incidence of psychosis in the UK ranges from 50.2 to 55 per 100 000 person years in East London^1^ and South East London, respectively, and 22–25 per 100 000 in Bristol and Nottingham.^2^ Patients presenting with psychotic disorders are usually young at their first episode, and may lack insight into their illness.^3^ Without prompt treatment they can experience poor quality of life and functioning, with more severe symptoms and a greater risk of violence.^4–7^ Early intervention for patients with a first episode of psychosis is therefore recommended to minimise duration of untreated psychosis and to avoid contact with the criminal justice system.^4,8^ Risk factors associated with violent behaviour may explain why criminal justice pathways (CJPs) are encountered as opposed to primary care pathways during first episodes of psychosis; for example, violence may be related to substance misuse, criminality and diagnosis associated with paranoia and poor insight, and these may be related to a longer duration of untreated psychosis.^2,9–11^ Furthermore, poor social support, few close confidants or not having a facilitative general practitioner (GP) can also lead to CJPs.^12,13^

Systematic reviews of CJPs have shown that they are more common among some ethnic groups.^14,15^ Of seven studies that investigated ethnicity and pathways to care, three found no ethnic differences in the pathway to care.^12,16,17^ Two British studies reported that African–Caribbean patients were more likely to have a CJP than White British patients.^18,19^ Furthermore, a recent study of early intervention services in London confirmed a higher risk of CJPs for Black Caribbean, Black British and Black African men.^8^ Our previous paper showed that CJPs were most common at the first pathway contact and were associated with a longer duration of untreated psychosis.^20^ The cause of a higher risk of CJPs among Black patients remains unexplained with inconsistent findings between studies.^21–23^ Although treatment delays cause more disturbed episodes of illness among African–Caribbean patients, and these were proposed to lead to a CJP,^1^ three London studies found no delays among Black ethnic minority groups.^8,20,24^ Personality difficulties, type of psychosis, criminality and substance misuse may all contribute to violent behaviour and may be related to ethnicity. Poor ‘insight’, where the patient's belief is that they do not have an illness and do not need treatment, can lead to escape behaviours, conflict and violence.^25^ Is the relationship between ethnicity and CJPs explained by more violence in some ethnic groups? Are there risk factors for both violence and CJPs such as substance misuse and psychopathy that might explain CJPs? This study investigates the extent of the aforementioned influences as mediators of the relationship between ethnicity and CJPs.

## Method

### Participants

The East London First Episode Psychosis study is a large, population-based incidence study conducted over 2 years in three neighbouring London boroughs of East London, England (City and Hackney, Newham and Tower Hamlets). Ethical approval was obtained from the local research ethics committee in East London. The detailed study design and sample characteristics have been reported previously.^1^

### Procedures

We identified all individuals aged 18–64 years living in the study area who made contact with mental health services because of a first episode of any probable psychotic disorder. After complete description of the study to the participants, written informed consent was obtained. The study took place over 24 months: from 1 December 1996 to 30 November 1998 in City and Hackney and from 1 December 1998 to 30 November 2000 in Newham and Tower Hamlets. All potential participants presenting to psychiatric services for the first time (including adult community mental health teams, in-patient units, forensic services, intellectual disability services, adolescent mental health services and drug and alcohol units) were screened. Health service bases were contacted weekly to identify all potential candidates. The initial inclusion criteria were based on those used in a World Health Organization (WHO) study and the Ætiology and Ethnicity in Schizophrenia and Other Psychoses Study (ÆSOP), although the lowest age for inclusion was 18 rather than 16 as in ÆSOP.^24,26^

To minimise leakage, methods used by Cooper *et al*^27^ were conducted during the study period to identify patients missed by the screening process, including checking with psychiatrists involved in private practice, private psychiatric hospitals served by the study area and high-security hospitals, reviewing new service registration forms in the medical records department and examining computerised information systems. Clinical professionals were contacted when there was uncertainty regarding potential participants. All patients who had been given a diagnosis of any psychotic syndrome were identified, and went on to subsequent stages of the protocol. Patients who passed the screening underwent a battery of assessments including the Schedules for Clinical Assessment in Neuropsychiatry (SCAN),^28^ the Personal and Psychiatric History Schedule^28,29^ and a schedule developed to record sociodemographic data. For all patients who could not be interviewed, the SCAN item group checklist was completed, based on case notes and information from clinical staff. Researchers were trained in the SCAN interview on a WHO-approved course to establish pre-study reliability using independent ratings of videotaped interviews. Diagnoses were allocated by consensus agreement between the principal investigator (J.W.C.) and the clinical researcher who conducted the individual assessments. The principal investigator remained masked to the ethnicity of the patient. Diagnoses were made using this and all other information from the case notes, item ratings in the SCAN and collateral histories according to the DSM-IV.^30^

Pathways to care were measured using the WHO pathways contact form, which documents the carer from whom help is initially sought (the first pathway contact) and then the next carer (second pathway contact) and finally a third pathway contact. This measure of pathways to care has previously been used in international studies of common mental disorders and psychotic disorders^11,31^ and studies of ethnic minorities in the UK.^32,33^ Services encountered in the pathway were described in accordance with the following categories: GP in primary care, accident and emergency departments, the police, community-based health and social care agencies including social workers and health visitors, prison services, psychiatric services, native or religious healers (traditional healers), hospital doctors (medical or surgical) and finally lawyers or the courts. The characteristics assessed included demographics, clinical and social characteristics that have been proposed to explain more CJPs.^18,19^ The questions at interview asked about presenting features such as violence (binary variable), any substance use over the previous year (as a binary variable including stimulants, opiates, solvents and cannabis), levels of alcohol use (units per week over the past year); a psychopathy checklist score of 12 or more (as a binary variable) that takes account of offending, risk-taking behaviour, lack of empathy and remorse;^34^ marital status (three-level variable: single; married or cohabiting; widowed, divorced or separated); social networks: number of people, education (any qualifications or none), employment (in paid employment or not) and place of birth (UK or not). We also asked whether the person was referred by someone else or self-referred for their first contact, thus reflecting awareness of illness as self-referral would indicate recognition of the need for treatment. Furthermore, we asked if the person had insight by enquiring whether they accepted they were ill, accepted they were ill but they did not question their delusional beliefs or did not accept they were ill (i.e. no insight).

Ethnicity was ascribed by a panel of researchers, one of whom was of African–Caribbean origin, using all available information, including self-ascription, place of birth and parental place of birth, the final decision being that of the researcher. In the study we coded ethnicity according to the same 16 categories used in the 2001 census. For analytical purposes, we collapsed the categories to produce nine ethnic subgroups: White British, White other (predominantly Irish and European), Black Caribbean (including Black other, mixed White and Black Caribbean groups), Black African, Indian, Pakistani and Bangladeshi. A category of all other ethnic groups (Chinese, other Asian and other mixed ethnic groups; *n* = 39) was included in the overall sample to ensure that pathway descriptions overall were of the total sample of people with first episodes of psychosis presenting within the defined period and in the defined geographical area. However, when ethnic group analyses were undertaken, this ‘other ethnic group’ category was not included as the findings would not be interpretable because of the heterogeneity of this group and analyses with such small numbers would be more liable to random error.

### Statistical analyses

For descriptive purposes, absolute (*n*) and relative frequencies (%) were reported for categorical variables, means and standard deviations (s.d.s) for variables on interval/ratio level. The findings of logistic regression models were presented as odds ratios (OR), a 95% confidence interval (95% CI) and a *P*-value showing the level of significance of difference when compared with the reference group. We combined first contacts for any of the criminal justice agencies (police, prison, courts/lawyers) to create a binary CJP outcome variable. We first assessed associations between ethnic group and CJP to identify which ethnic groups were at higher risk of CJPs compared with the White British reference group. Ethnic groups were then recoded to binary variables with White British as the reference group.

In order to identify the potentially mediating or explanatory variables for associations between specific ethnic group and CJPs, we then assessed associations between the specific ethnic groups and clinical or demographic variables, and associations between these clinical or demographic variables and CJPs. A potentially mediating or explanatory variables was identified if it was associated with both CJP and with the specific ethnic group that showed associations with CJP to *P*<0.05 level. By comparing standardised regression coefficients from models with and without violence at presentation as covariate,^35^ we estimated the proportion of direct effects that were mediated by violence and tested their significance using bootstrapped standard errors and confidence intervals (using 1000 repetitions). We estimated what percentage of the association between CJP and ethnicity was explained by each of the potentially mediating or explanatory variables. Mediation analyses were performed in Stata 11.0 using ‘binary mediation’ commands, and giving direct and indirect effect estimates, before and after including age, gender, marital status and employment as potential confounders. A significance level of *P*<0.05 was adopted throughout.

## Results

**Fig. 1:**
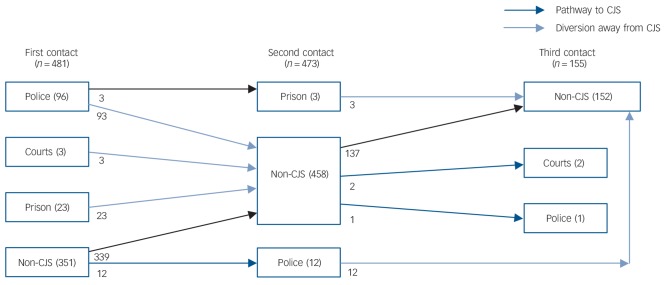
Pathways towards criminal justice system (CJS) for cohort of 481 patients. Ethnic breakdown by CJP for patients: police: White British 19, Black Caribbean 22, Black African 23; prison: White British 4, Black Caribbean 8, Black African 5; solicitor/courts: White British 0, Black Caribbean 1, Black African 0.

We recruited 295 men and 186 women, with a mean age of 30.68 years (s.d = 10.05, range 18–64). The majority were single (*n* = 278, 57.8%) or married (*n* = 101, 21%) and 214 (45.63%) had some qualifications; 241 (50.1%) were born in the UK. Table 1 provides the ethnic breakdown; the majority were of White British, Black Caribbean or Black African ethnicity (see Fig. 1 for overall pathways and for ethnic breakdown of CJPs).

**Table 1:** The association between ethnicity and criminal justice pathway (*n* = 122)^a^

Ethnicity	*n* (%)	OR (95% CI)	*P*
White British	23 (18.85)	1 (reference group)	

White other	14 (11.48)	1.03 (0.49–2.18)	0.94

Black Caribbean	31 (25.41)	2.97 (1.54–5.72)	0.001

Black African	28 (22.95)	1.95 (1.02–3.72)	0.04

Black other	1 (0.82)	0.77 (0.09–6.88)	0.81

Indian	4 (3.28)	0.73 (0.23–2.33)	0.59

Pakistani	3 (2.46)	0.88 (0.23–3.36)	0.86

Bangladeshi	4 (3.28)	0.26 (0.08–0.78)	0.02

Other	14 (11.48)	2.14 (0.96–4.77)	0.06

a. Logistic regression (unadjusted).

### Ethnicity and CJP

Compared with the White British Group, CJPs were more common among Black Caribbean and Black African patients, and less common among Bangladeshi patients (Table 1). However, only four Bangladeshi patients were in contact with CJPs and therefore they were excluded from subsequent analyses as the estimates would be imprecise.

### Factors associated with CJP

Table 2 shows the actual numbers of people and proportion of the total sample that have specific demographic, social and clinical characteristics (see online Table DS1 for a version of Table 2 that includes a wider range of characteristics). CJPs were more common among men rather than women, for violent presentations, for those who used drugs in the preceding year and for those with a higher level of psychopathy. CJPs were less common among women, married compared with single people, homemakers compared with employed people, those with a DSM-IV diagnosis of either schizoaffective disorder or affective psychosis (-depression) compared with those with a diagnosis of schizophrenia. Social factors including number of close people, a first referral by self or other people, alcohol use and insight were not significantly associated with CJPs; non-UK place of birth and having educational qualifications did not show statistically significant associations with CJPs.

**Table 2:** Associations of demographic and clinical variables with criminal justice pathway (*n* = 122)^a^

	*n* (%)	OR (95% CI)	*P*
Gender			
Male	97 (79.51)	1 (reference group)	
Female	25 (20.49)	0.65 (0.42–1.00)	0.05

Marital status			
Single	91 (74.59)	1 (reference group)	
Married	11 (9.02)	0.25 (0.13–0.49)	<0.001
Cohabiting	2 (1.64)	0.24 (0.06–1.07)	0.06
Divorced/separated	16 (13.11)	0.57 (0.31–1.04)	0.07
Widowed	2 (1.64)	0.59 (0.12–2.88)	0.51

Employment			
Employed	90 (75.63)	1 (reference group)	
Homemaker	2 (1.68)	0.14 (0.03–0.60)	0.01
Unemployed	6 (5.04)	0.62 (0.25–1.55)	0.31
Student	18 (15.13)	1.79 (0.95–3.40)	0.07
Other	3 (2.52)	1.67 (0.39–7.14)	0.49

Diagnosis			
Schizophrenia	53 (43.44)	1 (reference group)	
Delusional disorder	7 (5.74)	0.67 (0.27–1.67)	0.39
Brief psychotic disorder	16 (13.11)	1.13 (0.57–2.24)	0.73
Schizoaffective disorder	11 (9.02)	0.29 (0.14–0.59)	0.001
Non-affective psychosis NOS	8 (6.56)	0.81 (0.34–1.94)	0.63
Affective psychosis – depression	9 (7.38)	0.32 (0.15–0.70)	0.004
Affective psychosis – mania	18 (14.75)	1.23 (0.63–2.39)	0.55
Affective psychosis NOS	0	–	–

Violent at presentation to services	66 (54.10)	4.23 (2.74–6.54)	<0.001

Psychopathy, score 12 or more	24 (20.34)	2.55 (1.43–4.53)	0.002

Drug use in past year	71 (58.68)	2.28 (1.50–3.47)	<0.001

NOS, not otherwise specified.

a. Logistic regression (unadjusted). See online Table DS1 for a version of Table 2 including a larger number of characteristics.

### Identification of explanatory variables

Table 3 shows associations of demographics (e.g. age, gender) and potential explanatory or mediating variables in the Black Caribbean and Black African ethnic groups, but only for variables that showed associations with CJPs in Table 2 as only these might be potentially mediating or explanatory variables. Violence at presentation was associated with Black Caribbean ethnicity and CJPs therefore it might be a mediator for the relationship between Black Caribbean ethnicity and CJPs. However, violence was not associated with Black African ethnicity and so it cannot mediate the higher risk of CJPs for patients in the Black African ethnic group. Although a psychopathy score, including criminal activity, of ≥12 was associated with CJPs (Table 2), a psychopathy score of >12 was less common among Black African patients, and so it could not explain the higher risk of CJP contact for Black Africans. A lower psychopathy level in Black Africans indicates that the risk of CJPs would be even higher if the experience of psychopathy was the same as for White British patients and Black Caribbean patients. As this could not explain the absence of association between violence and Black African ethnicity, further mediation analyses of the Black African group were not undertaken. Drug use in the previous year was not associated with Black Caribbean or Black African ethnicity and so cannot be a potentially mediating or explanatory variable for associations between ethnic group (Black Caribbean or Black African compared with White British patients) and CJPs. Diagnoses were associated with CJPs, but not with ethnic group and therefore cannot operate as potentially mediating or explanatory variables.

**Table 3:** Associations of demographic and clinical variables with ethnicity^a^

	Black Caribbean^b^		Black African^b^	
	OR (95% CI)	*P*	OR (95% CI)	*P*
Age	0.98 (0.95–1.00)	0.13	0.96 (0.93–0.99)	0.02

Gender				
Male	1 (reference group)		1 (reference group)	
Female	1.32 (0.72–2.45)	0.34	1.03 (0.57–1.88)	0.92

Marital status				
Single	1 (reference group)		1 (reference group)	
Married	1.09 (0.41–2.91)	0.86	2.14 (0.91–5.00)	0.08
Cohabiting	0.90 (0.21–3.94)	0.89	1.47 (0.40–5.35)	0.56
Divorced/separated	0.65 (0.29–1.49)	0.31	0.77 (0.35–1.68)	0.51
Widowed	–^c^	–	–^c^	–

Employment				
Employed	1 (reference group)		1 (reference group)	
Homemaker	0.47 (0.90–2.39)	0.36	0.75 (0.18–3.12)	0.69
Unemployed	0.56 (0.17–1.87)	0.34	0.15 (0.02–0.12)	0.07
Student	0.93 (0.25–3.45)	0.10	5.00 (1.89–13.23)	<0.001
Other	–^c^		0.50 (0.05–4.93)	0.55

Diagnosis				
Schizophrenia	1 (reference group)		1 (reference group)	
Delusional disorder	0.88 (0.25–3.06)	0.84	0.76 (0.20–2.84)	0.68
Brief psychotic disorder	0.34 (0.09–1.32)	0.12	1.32 (0.50–3.48)	0.57
Schizoaffective disorder	0.76 (0.33–1.77)	0.53	1.07 (0.48–2.40)	0.87
Non-affective psychosis NOS	0.82 (0.21–3.18)	0.77	0.88 (0.23–3.42)	0.85
Psychotic depression	0.65 (0.26–1.60)	0.35	0.49 (0.18–1.31)	0.16
Psychotic mania	0.93 (0.29–2.96)	0.90	1.98 (0.71–5.50)	0.19
Affective psychosis NOS	–^c^	–	–^c^	–

Violent at presentation to services	2.65 (1.38–5.09)	<0.001	1.50 (0.78–2.89)	0.22

Psychopathy (score 12 or more)	0.71 (0.34–1.49)	0.36	0.12 (0.04–0.42)	0.001

Drug use in past year	0.87 (0.46–1.62)	0.65	1.02 (0.56–1.86)	0.06

NOS, not otherwise specified.

a. Logistic regression (unadjusted). Reference group.

b. Comparison group: White British.

c. No estimate possible because of sparse data.

### Mediation analyses

**Fig. 2:**
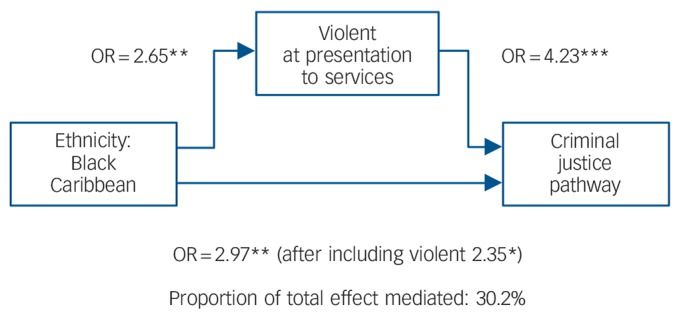
Mediation analyses for violence and criminal justice system (CJS) among Black Caribbean patients: logistic regression showing direct and indirect pathways. *P<0.05, **P<0.01, ***P<0.001.

Mediation analyses were undertaken for violence as the potentially mediating or explanatory variable for the Black Caribbean group (Fig. 2). The indirect effect was 0.0891, and the direct effect 0.2063, with a total effect of 0.2954. Violence mediated 30.2% of the effect between being Black Caribbean and having a CJP. When these analyses included age, gender, marital and employment statistics as potential confounders, the estimates did not change substantially with violence then explaining 31.13% of the overall effect and an adjusted bootstrapped estimate (1000 repetitions) was almost identical (31.12%) (Fig. 2).

## Discussion

This paper shows that violence at first presentation is associated with CJPs for people developing a new-onset psychoses; violence mediates some of the risk of Black Caribbean people with a new psychosis encountering CJPs, but certainly not all of it. Violence did not mediate the risk of Black African people with psychosis encountering CJPs, for whom a high psychopathy score (including questions on previous offending) was relatively less common compared with the White British reference group; so psychopathy could not explain a higher risk of CJPs. The Bangladeshi group appeared to have a lower risk of encountering CJPs but the actual numbers were too small to run mediation analyses.

Drug use in the previous year did not explain CJPs, and it was not associated with Black Caribbean or Black African ethnicity, and so does not meet criteria to be considered a potentially mediating or explanatory variable. Cannabis use before the age of 14 has been implicated in later psychosis in Trinidad^36^ and frequent cannabis use is associated with longer-term risk of psychosis, but not with ethnic patterns of psychosis.^37^ A meta-analysis also confirmed that drug use in psychotic populations was found to be more prevalent in forensic services, although again this does not explain ethnic patterning of pathways to psychiatric care through CJPs.^38^ CJPs appeared not to reflect social isolation or insight. The findings contrast with some studies showing that clinical and social factors account for a higher risk of CJPs in psychoses.^18,39^ Although studies of ethnicity and CJPs have been criticised for having inadequate numbers of social variables,^9^ from our present study, with a large sample size, social isolation still appeared not to explain the higher risk of CJPs.

The overall findings on CJPs for the Black Caribbean group contrast with the findings for the Bangladeshi group, which was less likely to have CJPs. The Indian and Pakistani groups showed no associations with CJPs. Previous studies of pathways in first-episode psychosis have not disaggregated South Asians groups, and so may have overlooked important differences. A recent systematic review reports no consistent findings about South Asians and CJPs.^15^ Larger samples of South Asian subgroups are needed to compare them more precisely, and if a lower risk is found, then this may give clues about how to explain and therefore reduce the risk of CJPs for other groups. Since the data were collected for this study, Asian groups in the UK have increased and in 2011 were estimated as 7.5% of the population (2.5% Indian and 2% Pakistani).^40^ The incidence of psychosis is also elevated in South Asians^41^ and so future studies, especially in urban areas, may be able to recruit sufficient numbers to replicate our analyses among South Asians.

Future research is needed into the causes of violence in first-episode psychosis, and preventive efforts may more closely focus on the role of substance misuse and detailed personality types. At the same time, the finding that CJPs are common at a first contact, suggest more health promotional information about psychosis may be important to encourage prompt referral to non-CJPs. Such a public health response might be targeted at those with family histories of psychosis or any mental disorder, and those high-risk social and ethnic groups. A public health response can be appropriately located and organised within a primary care setting.^42^ Criminal justice agencies and the police also need to ensure appropriate diversion at the earliest opportunity.

The police were the most common contact in CJPs. A recent inquiry in the UK investigated deaths in custody, and the role of the police when in contact with people showing symptoms of a mental disorder.^43^ This concluded that systemic and organisational changes had to take place, and that both the police and healthcare services need a shared emergency pathway, and to be better prepared to assess and manage people with mental disorders who are commonly in contact with CJPs. A lack of well-staffed and skilled assessment by the police was thought to contribute to tragedies, as did marked variations in practice in provider organisations in the National Health Service. Encouraging early diversion away from police contact, where the offences are not so serious that they require remand in custody, may also enable effective resource savings.

We have shown the role of violence as a mediating factor in CJPs for Black Caribbean patients, but we did not have access data on different offending behaviours that might potentially account for CJPs.^44^ We did include a measure of psychopathy, reflecting criminal behaviour, and this showed associations with CJPs, as did violence and drug use. However, psychopathy, and by implication criminal behaviour, were less and not more common in both Black Caribbean and Black African patients suggesting that criminality was not influential. And as the majority of patient were ultimately referred to non-CJPs, ethnic variations in offending may be less relevant in first-episode psychosis. A more recent study of first-episode psychosis in London found the same associations between CJPs and ethnicity,^8^ suggesting that the modernisation of mental healthcare and an array of community-based teams have not altered this association. Our findings investigated the causes of the association and may help to develop preventive interventions suited to particular contexts and groups to minimise CJPs.

### Strengths and limitations

The study relied on interview and self-report data that may be subject to recall bias. Questions are often raised about the validity of psychiatric diagnoses across ethnic and cultural groups.^45^ However, a strength of the study is that interviews were structured and questions were adopted from well-established, validated interview schedules and included the WHO SCAN for diagnostic assessment.^28^ This study, in an inner-city urban area, collected service, clinical and demographic information in a defined geographical area, and followed well-established methods.

### Future directions for research

Some groups remain at risk of CJPs, despite taking account of potentially mediating or explanatory variables, so further investigation is needed to discover and test new potentially mediating or explanatory variables. A combination of qualitative hypothesis generating and quantitative hypothesis testing studies are needed to better understand what influences the early contact with a CJP. This may be driven by illness perceptions and confidence in services, or stigma associated with services; or because of escape behaviours to avoid services leading to greater concerns about risk and more secure levels of care, or the nature of symptoms at presentation may be influential; these are not necessarily related to violence. The occurrence of violence and CJPs in psychosis remains an important research and clinical priority.
